# Multiple Chemical Sensitivity and the Workplace: Current Position and Need for an Occupational Health Surveillance Protocol

**DOI:** 10.1155/2013/351457

**Published:** 2013-06-16

**Authors:** A. Martini, S. Iavicoli, L. Corso

**Affiliations:** INAIL Research, Department of Occupational Medicine, Via Alessandria 220/E, 00198 Rome, Italy

## Abstract

Multiple chemical sensitivity, commonly known as environmental illness, is a chronic disease in which exposure to low levels of chemicals causes correlated symptoms of varying intensity. With the continuous introduction of new substances, people with MCS suffer significant limitations to their living environment and frequently to their workplace. This paper describes the current situation as regards MCS and the critical points in its case definition, which is still not generally agreed upon; this makes it difficult to recognize with certainty, especially, its precise relationship with work. Other problems arise in relation to the occupational physician's role in diagnosing and managing the worker with the disorder, the question of low levels of exposure to chemicals, and the best measures possible to prevent it. A diagnostic “route” is proposed, useful as a reference for the occupational physician who is often called in first to identify cases suspected of having this disease and to manage MCS workers. Work-related problems for people with MCS depend not only on occupational exposure but also on the incompatibility between their illness and their work. More occupational physicians need to be “sensitive” to MCS, so that these workers are recognized promptly, the work is adapted as necessary, and preventive measures are promoted in the workplace.

## 1. Introduction

Multiple chemical sensitivity (MCS), often referred to as environmental illness (EI), is an acquired chronic disorder in which exposure to low levels of chemicals causes related symptoms of varying intensity, from mild to totally disabling. Symptoms can affect multiple organs or systems: nervous, cardiovascular, gastrointestinal, respiratory, genitourinary and skeletal-muscular systems, skin, and ocular epithelia [1–4].

The etiology and pathogenesis of MCS is still not clear and it is hard to estimate its prevalence on account of numerous factors. For instance, (a) the various names given to the disorder and the fact that a single term can often comprise several pathological pictures mean it is difficult to find pertinent published studies; (b) there still seems to be no case definition accepted by all healthcare workers; (c) most reports do not list in full the criteria used to define cases; and (d) the various studies often use different diagnostic tools and investigation strategies (telephone interviews, hospital diagnoses, etc.).

Often the prevalence rates in the literature are “self-reported,” with substantial differences between the percentages of self-reported cases and those diagnosed by medical staff, particularly by occupational physicians. Between 13% and 33% of people in various populations consider themselves to be “unusually” sensitive to certain common environmental chemicals [4–11]. 

The literature review for the preparation of the *Documento de Consenso sobre Sensibilidad Quimica Multiple* (Consensus Document on Multiple Chemical Sensitivity, based on the best available scientific evidence, is intended to help healthcare workers make decisions on diagnosis, treatment, prevention, and other aspects of MCS) shows a difference between the percentage of people who consider themselves ill (0.48–15.9%) and those diagnosed by physicians (0.5–6.3%) [12]. 

In a US study in 2003 on a sample of the urban population of Atlanta, self-reported MCS was 12.6%, while medical diagnosis is found only 3.1% [13]. In a study a year later on the entire US population, the prevalence of self-reported MCS was 11.2%, while medical diagnosis gave a figure of 2.5% [9]. A study in Germany in 2005 found a prevalence of self-reported MCS of 9% while the prevalence from medical diagnosis was 0.5% [14]. 

Different data collection methods might partially explain the differences in prevalence of MCS. On the other hand, since MCS is underdiagnosed, it is probably more useful to rely on epidemiological surveys. 

In the US it is estimated that, respectively, 12%, 16%, and 18% of the local population in Atlanta, California, and North Carolina are particularly sensitive to chemicals [9, 10, 15]. 

A study by Caress and Steinemann in the US population found 11.6% of people reporting adverse effects from exposure to perfumed products [16]. A study funded by the Ontario Ministry of Health found that 3.1–6.3% of the Canadian population reported diagnosis of MCS [17]. 

A survey in Nova Scotia, Canada, showed that 3% of the Canadian population had had a diagnosis of environmental illness, but also that one in eight adults had complained of symptoms, gone absent from work, and complained of impaired ability to work due to exposure to “normally safe” levels of some common chemicals [18]. 

A Canadian Community Health Survey (2005) reported the prevalence of MCS in a target population (excluding Canadians living in institutions, native Canadians living on reserves, full-time members of the armed forces, and Canadians living in remote regions) by age and sex; the total prevalence was 2.5% of adult Ontarians, rising with age, and peaking at 5.8% in women between the ages of 60 and 64 years [11]. 

As regards the sex distribution of MCS, in all studies women were the most affected. Proportions were between 55 and 100%, with a mean of 81.5% [19]. Other studies too found a larger number of women with MCS, with 60.7% and 86.2% [9, 20]. Women may be more vulnerable because of exposure to chemicals at home and other indoor workplaces such as offices, hospitals, or schools. Even biological and hormonal differences make women more vulnerable. Many compounds in pesticides and plastics are endocrine-disrupting chemicals (EDCs) that can copy or imitate natural hormones. EDCs tend to accumulate in fat, interfere with the function of hormones in the body, and can cause other health problems, even at low levels of exposure [21]. 

## 2. Case Definition

The case definition has seen changes over time. In 1987, Cullen identified MCS as “an acquired disorder characterized by recurrent symptoms, referable to multiple organ systems, occurring in response to demonstrable exposure to many chemically unrelated compounds at doses far below those established in the general population to cause harmful effects. No single widely accepted test of physiological function can be shown to be correlated with the symptoms” [22].

In February 1996, the invited experts forming a workshop organized by the International Program on Chemical Safety (IPCS) of the WHO, the United Nations Environment Program (UNEP), and the International Labor Organization (ILO) recommended a new name: idiopathic environmental intolerances (IEI) because the term MCS “makes an unsupported judgment on causation” (i.e., environmental chemicals). This concept was taken from Sparks (2000), who defined IEI as a chronic recurrent condition, caused by a person's inability to tolerate an environmental chemical or a class of exogenous chemicals [23–26]. 

IEI, according to the proponents, is a complex gene-environment interaction, whose real cause is not known, for which it is possible—though not always—to identify a triggering event (e.g., sniffing a substance) and a response involving one or more organs or systems. Depending on its characteristics (i.e., the prevalence of somatic or psychological disorders) it can be confused with allergic reactions or psychiatric illness [27, 28]. 

However, multiple chemical sensitivities (MCS) is still the term most widely used to describe the complex syndrome; it presents as a chain of symptoms linked to a wide variety of environmental agents and components, at levels normally tolerated by most people [11].

The first attempt to establish some criteria for the standardization of the symptoms and their classification was proposed in 1987 and has to do with the compatibility between symptoms and exposure to chemicals, the supposed relationship between exposure and the onset of symptoms, and the exclusion of other known diseases. Lax and Henneberger (1995), analyzing the data for hundreds of individuals considered to have MCS, showed that only 6.4% met the diagnostic criteria of Cullen [29]. 

Currently, the most widely adopted criteria for the recognition of MCS are proposed in a consensus document [30, 31]:

1. the symptoms are reproducible with (repeated chemical) exposure;
2. the condition is chronic;
3. low levels of exposure (lower than previously or commonly tolerated) result in manifestations of the syndrome;
4. the symptoms improve or resolve when the incitants are removed;
5. there are responses to multiple chemically unrelated substances;
6. symptoms involve multiple organ systems (added in 1999).

This international document, published in 1999, was the product of a multidisciplinary study conducted by 89 clinicians and researchers with broad experience in the field. There were 36 allergologists, 23 occupational physicians, 20 clinical ecologists, and 10 internal medicine and ENT—ear, nose, and throat specialist, with the aim of establishing a case definition for MCS.

In 2005 Lacour et al. [32] proposed extensions to the definition criteria, including the following:

1. chronic condition lasting more than six months and causing deterioration of lifestyle and body functions;
2. symptoms recur reproducibly and affect the nervous system, with a characteristic hypersensitivity to odors;
3. continuous involvement of the central nervous system and of at least one other apparatus;
4. responses induced after low levels of exposure;
5. responses to multiple unrelated chemicals;
6. improvement or resolution after removal of exposure.

The wide range of symptoms with which MCS manifests and the difficulties of differentiating them from other pathologies—immunologic, digestive, cardiac, respiratory, psychiatric, neurologic, endocrine, and so forth—make it hard to develop a diagnostic tool that specifically identifies patients with MCS. The 1999 Consensus Document suggests using the Environmental Exposure and Sensitivity Inventory (EESI) to investigate patients for MCS. The authors subsequently modified this for faster, more widespread use, as the Quick Environmental Exposure and Sensitivity Inventory (QEESI). Some investigators have used the questionnaire in its original form but modified or adapted to take account of geographical differences [3, 33–41].

The QEESI was developed as a screening questionnaire for multiple chemical intolerances (MCI). The instrument has four scales (symptom severity, chemical intolerances, other intolerances, and life impact) and can be used for the following applications:

1. research: to characterize and compare study populations and to select subjects and controls;
2. clinical assessment: to obtain a profile of patients' self-reported symptoms and intolerances; patients can be asked to complete a QEESI at intervals in order to follow the course of their illness over time or in response to treatment or exposure avoidance;
3. workplace or community investigations: to identify and provide self-assessment information to individuals who may be more susceptible or who report new intolerances; affected employees should have the opportunity to discuss the results with investigators or their personal physicians.

A simplified version of this questionnaire was employed in a study by Fabig for screening MCS patients. The first part focuses on the type(s) of substance with which the patient might have contact and the intensity of the disorders present. The second part examines the type and severity of the disorders the patient suffers after exposure to the culprit substance(s).

In the original QEESI there were from 0 to 10 responses for each substance. The modified version avoids this “excessive detail of subjective evaluations,” with three possible answers to each question on the level of the disorders related to ten types of exposure. The minimum score in the QEESI modified according to Fabig is 10 (no disorder), and the maximum is 30 (serious disorders after exposure to all the substances listed). A score between 10 and 20 indicates a normal situation, while 21–30 suggests MCS [42].

There are other questionnaires too, to help in diagnosis. One of these is the University of Toronto Health Survey (UTHS) which starts with various case definitions for MCS. It then identifies a series of symptoms related to low-dose exposure. The reproducibility of the UTHS is evaluated in relation to the seven case definitions. Another diagnostic aid is the Idiopathic Environmental Intolerance Symptom Inventory (IEISI) which investigates the frequency of symptoms in MCS patients [22, 30, 37, 43–46].

Other questionnaires investigate the severity of the environmental chemical sensitivity [8, 11, 36, 47–49]. 

## 3. MCS and Work

### 3.1. Work-Related MCS and Workers with MCS

A definition of work-related MCS was introduced in 1987 [22]. This definition, subsequently amended, includes a number of health effects observed in workers who had been exposed to low levels of different chemicals. With the continuous introduction of new substances, both indoors and outdoors, people with MCS suffer significant limitations in their living environment [50] and frequently in their work environment.

There are more than 70 million unique chemical substances, organic and inorganic, on the market, such as alloys, coordination compounds, minerals, mixtures, polymers, and salts. Every day about 15,000 new substances are added [51]. The chemicals are found in many products of daily use, such as detergents, textiles, clothing, and furniture. They are employed not only by workers in the industries that produce them, but also—widely—in other industries: construction, metalworking, woodworking, automotive, textile, food, agriculture, information technology, waste management, cleaning, and so forth.

The work-related problems for people with MCS do not depend only on occupational exposure but also on the incompatibility between their illness and their work [52]. A study of people with self-reported MCS found that three-quarters of the 268 respondents had lost or had had to leave their jobs because they did not tolerate exposure to chemicals present in the environment. The Human Ecology Action League (HEAL) survey of 269 people with MCS showed that 45% had lost their jobs [53]. Caress and Steinemann found that 1.8% of their random community sample of 1582 people had lost their jobs on account of hypersensitivity to common chemicals [12].

### 3.2. Categories of Workers at Higher Risk

It is usual to define as “sensitive” an individual who responds adversely to low exposures to chemicals. While a person sensitive to chemicals can be found in any group, a classification of the job categories most at risk has been attempted several times. At first, in studying the relation between MCS and work, attention focused mainly on patients who were industrial workers, initially suggesting that MCS may be linked to occupational, therefore potentially intense, chemical exposure. A subsequent study by Cullen and coworkers of all MCS patients seen at Yale University Occupational Medical Clinic between 1986 and 1992 found only low rates of MCS in industrial sectors, associated with the highest rates of chemical and physical injuries. Only about 27% of patients with MCS were occupationally exposed to chemicals present in the construction and manufacturing sectors, paradoxically suggesting that exposure backgrounds with low levels of chemical exposure are more likely to be associated with MCS than those with high exposure [54]. 

After some time, similar problems were described in occupants and workers in “tight” (tightly closed) buildings, residents of communities whose air and water were contaminated by chemicals and persons who had experienced personal exposure to various chemicals in domestic indoor air [55]. The classification of Ashford and Miller was further amended and supplemented by Winder [56], as shown in Table 1, outlining the exposure conditions and demographics:

- workers who are occupationally exposed to chemicals as part of their everyday activities;
- employees who work in tightly closed buildings;
- individuals working in contaminated areas;
- people who, for one reason or another, were unexpectedly exposed to a chemical substance.

The authors described various demographic characteristics of these groups. For example, industrial workers are predominantly male, whereas those with chemical sensitivity from tightly closed buildings and those with “personal and unique” chemical exposures are a heterogeneous group, though predominantly female, white-collar, or professional.

Similarly, of 200 individuals with MCS (case definition not mentioned), observed at an environmental health center in Dallas, USA, less than 5% were workers, and the highest percentage (25%) were housewives, suggesting an association between certain domestic chemical exposure events and MCS. Just like the demographic findings of other studies, most of these MCS patients were women, who presented themselves for examination mainly at an age of around 30 to 40 [57]. 

Lax and Henneberger in 1995 [29] identified 35 of the 605 new patients who presented for visits between 1989 and 1991 as meeting a case definition similar to that proposed by Cullen [22]. In this study, 54% of the non-MCS patients had worked in sectors considered to be at greater risk of dangerous exposure to chemicals than other workplaces. In contrast, only 26% of patients with MCS were employed in the more risky sectors [58].

The US Environmental Protection Agency (EPA) reported that about one-third of people employed in a closed work environment reported particular sensitivity to one or more common chemicals [59]. In fact, supporters of the existence of MCS have described a greater spread among women, aged between 25 and 50, who spend many hours inside sealed or otherwise closed buildings, those living and working in cities with high pollution, and among the users of deodorants, perfumes, detergents, insecticides and herbicides [9]. 

In the work environment, Watanabe et al. identified as at-risk categories users of chemicals, especially volatile compounds such as organic solvents, or workers belonging to certain categories such as farmers, construction workers, urban policemen, and hairdressers, but especially housewives [60]. Lucchini et al. identified the professional categories most frequently affected by the syndrome as workers in industry in general (where it is easier to come into contact with chemicals) and in particular where solvents are used, as well as farmers, construction workers, policemen, hairdressers, housewives, and office workers [61]. A 2008 study examined pest controllers frequently exposed to pesticides, a class of chemicals commonly associated with MCS. There was an increased risk of development of MCS in this category [62]. 

In 2005, a South Australian parliamentary inquiry into MCS collected data from healthcare professionals caring for patients suffering from the disorder, which showed the role played by certain chemicals such as detergents, glutaraldehyde, and formaldehyde in triggering MCS. In support of this theory, in 1998 a national support group was established for individuals who suffer from health problems apparently related to exposure to hazardous chemicals in the workplace, including glutaraldehyde. So the Glutaraldehyde-Affected Support Persons Injured Nurses Group (GASP-ING), which evolved primarily as a network of shared experience, identified glutaraldehyde as a chemical of particular concern for healthcare workers [58]. 

The oils and hydraulic fluids used in aircraft engines can be toxic, and specific ingredients of oils can be irritating, sensitizing, and neurotoxic. In fact, flight crews and cabin crews have identified exposure to engine oil or hydraulic fluid leaks as a cause of their diagnosis of MCS [63].

A report from the Danish Environmental Protection Agency in 2005, that reviewed the state of knowledge regarding MCS, reported cases in Denmark among people exposed to organic solvents or pesticides at work [64]. A group from the Division of Environmental Medicine in Stockholm reported a higher frequency of MCS-like symptoms among housepainters than other job categories [65]. In a Danish study, two cases of MCS were described among workers employed in public swimming pools. Chlorine vapors, which are formed in special circumstances, caused the onset of symptoms (e.g., trihalomethanes and chloramines). In both cases, the patients had to leave work and go through reeducation. The Danish consensus document indicates that a wide range of people from different professional groups display symptoms of MCS: healthcare, aviation, farmers, mechanics, and aluminum workers at Alcoa Wagerup [64]. 

An analysis of the literature shows that there are numerous categories at risk of developing MCS (Table 2).

### 3.3. Chemicals Related to MCS

Many substances have been called into question in the onset of MCS. It is difficult to classify them comprehensively and systematically, partly because of the continuous introduction of new chemicals on the market. There are, however, numerous extensive lists in several publications and articles.

Ashford et al. in the European report of 1994 proposed a classification of chemical compounds associated with MCS, grouped according to the source of exposure [66]:

- external contamination: pesticides, volatile solvents, and paint fumes;
- fuels, combustion products, tars, emissions from diesel and gasoline engines and air of industrial areas;
- indoor air pollution at home and at work, especially in confined spaces: products of gas combustion and domestic heating, synthetic sponges, plastics, pesticides, perfumes, deodorants, detergents, cleaning products, disinfectants, ink of newspapers and other printed materials, fabrics, curtains, rugs, odors of petroleum derivatives, wood, and cooked food;
- food additives and contaminants, such as corn and sugar, residues of pesticides, fungicides, artificial colors, preservatives, food sweeteners, protective waxes, and packaging materials;
- water contaminants and additives ingested in drinking water;
- drugs and consumer products such as aspirin, barbiturates, sulfa drugs, diluents, flavorings, preservatives, mineral oils, lotions, laxatives, synthetic vitamins, adhesive tape, cosmetics, perfumes, shampoos, personal hygiene products, dental adhesives, salts and bath oils, water beds, pens, polishes, chlorinated pools, radiographic contrast medium, contact lenses, plastic components, and medical equipment.

Ziem in 1999 identified substances that can cause MCS generally after repeated exposure to low doses: pesticides, solvents, combustion products, renovating “sick” buildings, carbonless copy paper, other irritants, and petrochemical products [67]. 

In the USA in 2003 a list of twelve chemicals that trigger symptoms was published—substances which in a population study were most frequently associated with MCS. The list of chemicals included cleaning products, perfumes, pesticides, traffic fumes, products used in beauty and hair salons, carpets, furniture, chlorine in drinking water, and fresh ink markers [19]. 

In general, perfumes are frequently signaled as chemical compounds of interest (82.5%), followed by tobacco smoke, new housing, pesticides, petroleum products, fumes from combustion engines, and other chemicals [68].  Table 3 lists the agents recognized as related to MCS proposed in the Spanish Consensus Statement of 2011 [69]. 

A new group of people were described only a few years ago, with particular symptoms associated with exposure to electromagnetic radiation in connection with the use of electromagnetic devices. There is currently no scientific evidence that these symptoms are related to MCS [12, 27]. 

## 4. Primary and Secondary Prevention of MCS at Work

As the etiopathology of MCS is still not clear, the most effective approach to manage the disorder appears to be avoidance of triggering factors or situations in which the problem might arise. Consequently the reduction of environmental chemical exposures, particularly to pesticides and petroleum derivatives, is extremely important to reduce episodes of the disease. As this type of exposure seems to be associated with MCS, its reduction could have a wider preventive impact, for the general population too. Many of the chemicals involved have doubtful social utility, so reducing their use is anyway recommendable [11, 70]. 

The European legislation on chemicals has been “updated” and involves an integrated system of registration, evaluation, authorization, and restriction of chemicals—REACH—with the aim of protecting human and environmental health, and the European Chemicals Agency now deals with everyday questions relating to the REACH requirements, but it is still proving difficult to revise the exposure levels accepted to date [71].

It would be useful to have further knowledge of MCS and possible preventive approaches to support the coordination of national health surveillance projects and control of the use of chemicals as a result of application of this legislation throughout Europe. The findings of biomonitoring programs on persistent organic compounds could be integrated with data on the levels of human exposure to numerous other chemicals and the exposure situations that come to light from MCS research.

The main triggers may involve single exposure to high doses or multiple exposures to one or more substances. It is not always simple to verify the latter situation because the people concerned cannot always reconstruct their personal and/or occupational history. In addition, exposure may arise in different circumstances—at work, at home, accidents, food, and so forth. Since the risk of exposure to dangerous substances is theoretically universal, the persons involved would have to be isolated in order to avoid it. Clearly, this is not often feasible because it would be incompatible with work and daily life. Then too, with our still limited knowledge in this field, we have no means of creating public spaces where people would be fully protected from exposure.

Since it is prudent to avoid reexposure to triggering factors, people are advised to modify their usual habits, ventilating their premises thoroughly at home and at work, avoiding damp places, avoiding exposure to irritants such as gas or vapors, and following an appropriate diet, as far as possible. Avoiding exposure in daily activities and at work and changing lifestyle as necessary are in fact more effective measures than any therapy. Undeniably, however, these measures may limit a person's relations with other people, their access to work, and recreation.

At work, establishing an environment suitable on the whole for someone's health may require significant changes, which may need the cooperation of the people responsible for prevention—the occupational physician and occupational psychologist.

If the safety and prevention officers at work can identify the source of the problem at an early stage, this may be helpful in preventing the sensitivity mechanism from spreading and becoming chronic. It will be their task, with the occupational physician, to assess any adaptations or job change, considering the persons with MCS as sensitive workers. Workers who suspect they have MCS symptoms or their symptoms have got worse after exposure at work should therefore always consult the occupational physician [11]. 

An American study examined 605 patients at the Central New York Occupational Health Clinical Center in Syracuse, New York, between 1989 and 1991, to identify any who had a possible, probable, or definite diagnosis of MCS; 7.9% had a diagnosis, and the criteria for admitting them as MCS patients in the study required them to be defined as MCS cases, with evidence of exposure in the workplace. The identification of the pathology as work related relied on the clinical judgment of the physician who had examined the patient was based on several criteria: onset of symptoms following specific exposure in the workplace, worsening of the symptoms at work improvement when not there, no evidence of significant exposure outside the workplace, and symptoms among workmates [29].

People with MCS/EI may present some limitations, varying in severity from one individual to another. Not all of them need to make adaptations in order to work; some may only need minor changes. Adaptations may involve the ventilation system and quality of indoor air, the lighting, or aspects of the building, renovation work, and cleaning in the premises [72].

For example, in June 2009, the CDC put on its internal website an Indoor Air Environmental Quality Policy intended to maintain good indoor air quality in buildings where its employees work. Among other things, the policy states that scented or fragranced products are prohibited at all times in all interior space owned, rented, or leased by the CDC. This includes the use of the following products:

1. incense, candles, or reed diffusers;
2. fragrance-emitting devices of any kind;
3. wall-mounted devices, similar to fragrance-emitting devices, that operate automatically or by pushing a button to dispense deodorizers or disinfectants;
4. potpourri;
5. plug-in or spray air fresheners;
6. urinal or toilet blocks;
7. other fragranced deodorizer/reodorizer products.

Personal care products (including colognes, perfumes, and essential oils) should not be applied at or near actual workstations, restrooms, or anywhere in CDC-owned or leased buildings. In addition, the CDC encourages employees to be as fragrance-free as possible when they arrive in the workplace. Fragrance is not appropriate for a professional work environment, and the use of some products with fragrance may be detrimental to the health of workers with chemical sensitivities, allergies, asthma, and chronic headaches and migraines [73].

According to “A Guide for the Workplace” by Sine et al. a “no-scent” policy includes perfume, cologne, and after-shave and scented personal care products such as deodorants, shampoos, hair products, cosmetics, soaps, hand creams, laundry detergents, and fabric softeners. Smoke-laden and dry-cleaned clothing must be aired well before wearing. Avoid scented laundry detergents and all fabric softeners [74].

The CDC Indoor Air Quality Policy is a very important document and provides an example of what we should be doing in every workplace. All workplaces should be fragrance-free. The number of people who are chemically sensitive and/or have been diagnosed with MCS is increasing daily. This problem is very similar to those faced by workers when smoking was allowed in the workplace. The implementation of a smoke-free workplace policy by the Occupational Safety and Health Administration and other regulatory agencies has been very important in preserving the health of workers in those workplaces.

A fragrance-free policy allows individuals who are chemically sensitive to continue their employment. As a result, they do not have to turn to Social Security Disability for income. Those who are not the beneficiaries of a fragrance-free policy are often unable to work and do find themselves on Social Security Disability [73].

## 5. The Occupational Physician's Role in MCS Risk Management

In accordance with current European regulations, the occupational physician cooperates with the employer and the prevention and protection service in assessing risk in the workplace; when necessary this may involve establishing and implementing general health measures to safeguard workers' health and safety.

The general measures for this purpose include health surveillance, which the physician must plan and carry out in relation to the specific risks found in each workplace on the basis of the risk assessment, and in the light of the latest scientific information. Since it is the occupational physician who cooperates in drafting a risk assessment report—with its implications for health surveillance—his/her contribution is particularly important in assessing chemical risk. Often this type of risk assessment is based on algorithms and software that automatically quantify the secondary risk deriving from the use of dangerous substances (chemical risk) but which overlooks some of the fundamental principles of occupational medicine.

Software and algorithms can take into account numerous variables to establish how “important” a risk for health is: the amounts used, acceptable limits, and so forth. Some substances or classes of chemicals, however, need closer analysis because they can have an effect—causing harm—with no dose relation. The occupational medicine physician's contribution to any assessment of chemical risk is therefore a basic starting point, particularly as regards the idea of “low doses.”

The dose, or concentration, of a substance is considered low when—unlike a high dose—it has no toxic effects, is not measurable (it is below the level of detection), or does not significantly differ from the values found in the nonexposed population (reference value); it may be well below the limits allowed in a workplace. 

The relation between dose and effect, or response, is the basic principle for assessing toxic effects in general and those of chemicals in particular. The effect, however—or better, the probability of the effect (the risk)—does not depend solely on exposure, where the dose or concentration is the main quantitative variable; it depends on two other variables—the type of risk factor and the individual susceptibility of the person exposed. In other words some people may show high susceptibility to a certain chemical, either genetic or acquired, and cannot be protected even from low- or very low-dose exposure, for acute or chronic effects.

Hypersusceptibility causes an abnormal reaction to concentrations of a substance that would have no effect in most people; most members of a population would show no change in their health after exposure to a certain concentration of the substance, but a small proportion would suffer health problems—of varying severity—when exposed to the same concentration. Ideally, the occupational physician's checkup before someone is hired should aim to find out whether the candidate has any conditions and/or contraindications to the work proposed (e.g., hypersusceptibility) so that workers can be placed in an environment suited to their physiological and psychological capacities. Another aim of the prehiring checkup is to see whether the candidate is fit for the work proposed without posing any risk to him/herself or others [75].

When a person starts a job s/he must be questioned to detect any congenital or acquired conditions that might influence his/her susceptibility in the specific job. It is not a good idea to employ only hyposusceptible workers in a polluted workplace instead of taking all possible measures to clean up the environment; efforts should be made in the exact opposite direction—try to reduce the pollution so that even hypersusceptible people can work there.

It is a widespread idea in occupational medicine that the physician has detailed knowledge of each job—the work done, work cycles/shifts, workplaces, procedures, machinery, equipment, chemicals employed, and so forth. S/he must also be familiar with the workers' health and must have the tools to protect it. The occupational physician must assess a worker's clinical situation at the work station and judge whether that person is fit for the specific task, so as to safeguard workers with contraindications to the tasks required of them. Should the physician decide that a worker is not fit, on account of a disorder that has just become evident, s/he is obliged to assess whether the pathology is related to occupational exposure and, therefore, whether it calls for reexamination of current preventive measures [76].

Formulating a hypothetical diagnosis of MCS related to occupational conditions is part of this examination, but the facts that there is still no agreed definition of the disorder and MCS is not even universally recognized are important barriers to any evaluation. The following criteria, however, may be useful for formulating a diagnosis: (1) symptoms start after specific exposure in the workplace; (2) the symptoms become worse during work and may improve when the person moves away from the source of exposure; (3) there is no significant evidence of exposure outside work; and (4) colleagues at work show none of the symptoms [29].

One of the occupational physician's constant worries is that a worker will develop functional alterations that mean s/he cannot do the job; this calls for an opinion on the worker's temporary or permanent unfitness for the work. It is worth recalling that a person with MCS may usefully be moved temporarily from the job or given an alternative job for a short while; it is equally important, if s/he shows improvement, to give the worker a chance to return to work, if necessary with retraining.

The occupational physician faced with the possibility of a diagnosis of MCS may wish to work on the following considerations:

1. MCS is caused by occupational exposure: MCS subjects should be overrepresented in occupations with high relevant exposure (e.g., neurotoxic);
2. MCS is not caused by occupational exposure: the occupational distribution of MCS subjects should not differ from the general working population;
3. MCS is a chance effect, caused by strictly individual health problems: the occupational distribution of MCS subjects should not differ from the general working population;
4. MCS is an (acquired, genetic) susceptibility effect: the occupational distribution of MCS subjects should not differ from the general working population (unless susceptibility determines choice of career);
5. MCS is an oversensitivity plus selection effect (e.g., an accumulation of oversensitive individuals in underexposed intellectual occupations) MCS subjects should be underrepresented in occupations with high relevant exposure (e.g., neurotoxic);
6. MCS is a selective perception effect (e.g., different attitudes about “complaining” of exposure): the occupational distribution of MCS subjects should differ from the general working population, without MCS correlating with exposure.

As we have already noted, the MCS subject/worker develops symptoms at exposure levels well below official limits for the workplace, and sometimes in response to substances or preparations used, or commonly present, in any workplace's new furniture, cleaning products, printed paper, fragrances and perfumes, and so forth. The occupational physician must therefore find out what substances and preparations are used, based on information gained during scheduled inspections of workplaces, from prevention and protection services, and from talking to individual workers. S/he should also—as the CDC policy recommends—ask to be informed of the introduction of new chemicals in the work cycle, before they start to be used and of “renovation work or installation of new equipment” before it is done in the workplace. This practice would enable people with MCS and other chronic diseases such as asthma, allergies, and chronic obstructive lung disease to make other arrangements while the renovation work is going on, or the new carpeting, for example, is being laid. It also would allow them to discuss with their supervisors new chemicals that are being introduced into the work environment because often people who are chemically sensitive know about alternatives that are less toxic, not only for them but also for other people who may be affected [73].

Hygiene and safety rules at work do not only include health surveillance as one of the general measures for protecting workers' health, but expect it to be preceded by measures that give priority to eliminating or reducing risk [11].

### 5.1. Workers with MCS: Occupational Health Surveillance Protocol

The occupational physician's main responsibility is, as we have already said, to assess each worker's clinical status and, as required by local regulations, to establish a protocol for health surveillance. Its main aim would be to verify that there are no contraindications to a worker doing the job he is expected to do, to formulate a judgment that the worker is fit for that specific task, and to keep an eye on workers' health in the longer term. The physician has to plan and conduct this health surveillance following health protocols drawn up to take account of the specific risks in each case, in the light of the latest scientific knowledge.

Traditionally, the application of medical principles was a static process, modified on occasion by the practitioner's experience. In recent years, however, partly fuelled by computer-accessible databases, techniques for systematic review of clinical guidelines and economic analyses have become more widespread. For a variety of reasons, these evidence-based methods have only recently been applied to occupational health risks and interventions. As noted by Carter, the application of these methods in occupational medicine would likewise “improve the quality of prevention and would also enable practitioners to give more soundly based advice and to secure their professional positions as providers of quality-assured information” [77].

The analysis of the literature shows there is no single test yet that has proved 100% effective in diagnosing all MCS patients. MCS also does not meet the classic definition of an occupational disease which establishes a link between a specific condition and a specific exposure, as is the case of asbestosis and asbestos exposure. In fact exposure to a wide variety of substances can trigger a broad range of symptoms in MCS sufferers. A different approach must therefore be used to study the disease which is why there is no single known symptom or any definite test to establish an MCS diagnosis. The only sure element a physician can rely on is the person's environmental and medical history, combined with routine laboratory tests, which are intended to exclude other diagnoses [11].

**Figure 1:**
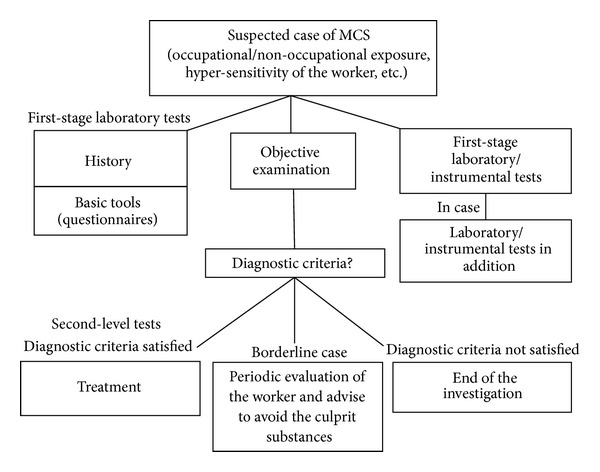
Flow chart-proposal of diagnostic protocol.

We outline a diagnostic strategy here for MCS, which may be useful to occupational physicians (Figure 1). 

Starting from the suspicion of MCS, based on various indications, episodes of exposure at work or outside, and/or hypersensitivity, the worker's history is collected in a detailed interview aimed at getting information on the chemical features of the environment where the disorder first presented itself, the setting where it developed, and the (work or personal) environment where the worker now spends his/her time; data must be recorded on environmental exposure times to chemicals, odor producing or not, indoors (household cleaning products, fitted carpets, etc.) and outdoors (nearby industrial plants, potential sources of pollution, etc.). Note when the indicative signs or symptoms appeared using a specific, detailed questionnaire, and record any adverse reactions to foods or drugs.

The objective examination must focus in particular on the worker's organs and systems related to the symptoms reported and on any signs that appeared subsequent to the exposure to chemicals. 

Basic tools in this phase are questionnaires (QEESI, UTHS, IESI, etc.), to be used before verifying that recognized diagnostic criteria are met. Tests may then be scheduled on the basis of each individual's history, the objective findings and/or any suspicion of associated pathologies, so as to exclude any other disorders. 

The following may be useful first-stage laboratory tests:

- complete blood count;
- serum iron, transferrin and ferritin;
- glycemia;
- immunoglobulins (IgG, IgM, IgA, and IgE);
- proteinemia and protein electrophoresis;
- electrolytes (Na, K, Cl, and Mg);
- lymphocyte typing;
- inflammatory indices (ESR—erythrocyte sedimentation rate, CRP—C reactive protein);
- liver function (GOT—glutamate oxaloacetate transaminase, GPT—glutamate pyruvate transaminase, gamma-GT—gamma-glutamyl transpeptidase, total and fractionated bilirubin total cholesterol, LDL—low density lipoprotein, and HDL—high-density lipoprotein, triglycerides);
- renal function (creatininemia, BUN—blood urea nitrogen).

In addition to laboratory tests, it may be useful to do an instrumental test such as global spirometry, which is fairly frequently used in occupational medicine.

Other tests may be added, on the basis of the worker's history, the objective findings, and the indications of each case:

- protein kinase C and isoenzymes;
- thyroid function (FT3—free triiodothyronine, FT4—free tetraiodothyronine, TSH—thyroid-stimulating hormone, antibody antithyreoperoxidase, antibody antithyroglobulin);
- blood coagulation picture (PT—prothrombine time, aPTT—activated partial thromboplastin time, fibrinogen, and homocysteine);
- antibody picture (ANA—antinuclear antibody, ENA—extractable nuclear antibody, antids-DNA—antidouble-stranded deoxyribo-nucleic acid, AMA—antimitochondrial antibody, ASMA—anti smooth muscle antibody);
- hepatitis virus markers (HbsAg—hepatitis B surface antigen, anti-HCV antibodies—antihepatitis C virus antibodies, and anti-HAV antibodies—antihepatitis A virus antibodies);
- serum tests for CMV—Cytomegalo virus, EBV—epstein-Barr virus;
- VDRL test—Venereal Disease Research Laboratory;
- urine tests and urine culture;
- screening for celiac disease (antiendomysium, antitransglutaminase, and antigliadin antibodies);
- breath test (urea, lactose, and lactulose);
- glutathione transferase and catalase activities;
- vitamins B1, B6, B12, folates, vitamin C, D3, E, and coenzyme Q10.

Second-level tests are indicated when the criteria such as the following are met:

1. suspicion of a history of MCS but no other pathologies that might explain the patient's symptoms;
2. substantial impairment of activities of daily life as a result of exposure to the chemicals;
3. two or more organs or systems affected after exposure to known chemicals;
4. questionnaire scores 21 or higher (the QEESI version modified by Fabig is recommended for its high sensitivity and specificity).

The following second-level tests may be useful:

- psychological assessment: personality questionnaires, self-assessment of symptoms of mental distress and quality of life (MMPI 1—Minnesota Multiphasic Personality Inventory 1, MMP2—Minnesota Multiphasic Personality Inventory 2, Rorschach test, and Zulliger test);
- neurophysiologic test: reaction times (simple and/or selected), balance, visual contrast, color, and vibration tests;
- allergy tests: skin reaction, patch test, specific and total IgE, eosinophilic cationic protein (ECP), and tryptase; oral exposure to drugs, foods, and additives; hypoallergenic diets;
- genetic polymorphisms: tests for genes implicated in oxidation (PON1—paraoxonase 1, CYP2D6—cytochrome P450 2D6, NAT2—N-acetyltransferase 2, GSTM1—glutathione S-transferase M1, GSTT1—glutathione S-transferase theta 1, GSTP1—glutathione S-transferase P1, and CAT—catalase) and assay of plasma proinflammatory cytokines;
- assays in biological samples to check for chemicals, metals, and/or their metabolites;
- metabolism and detoxification investigations.

If the worker fulfils the diagnostic criteria and further tests confirm the diagnosis of MCS, s/he should be sent for treatment. If it appears to be a borderline case, when the criteria are not all met but the clinical history suggests the problem may be there, further investigation is indicated, and the worker should be advised to avoid the culprit substances [11, 78, 79].

## Figures and Tables

**Table 1:** Classification of exposure conditions and demographics by Ashford and Miller (amended and supplemented by Winder).

Group	Nature of exposure	Demographics
Industrial workers	Acute or chronic exposure to industrial chemicals	Primarily males; 20–65 years old

Office workers (in ‘‘tightly closed buildings”)	Inadequate ventilation. Offgassing from construction or refurbishment materials or from office equipment. Tobacco smoke	More females than males. White-collar workers. 20–65 years old. School children

Contaminated communities	Toxic waste sites. Contamination by nearby industry sites. Aerial pesticide spraying. Groundwater contamination. Other community exposures	Low to middle classes. All ages, male and female. Children or infants affected first or most; possible effects in pregnant women

Individuals	Heterogeneous. Indoor air (domestic). Pesticides, consumer products, and drugs	White middle to upper classes, primarily females, 30–50 years old

**Table 2:** Categories at high risk of MCS.

Industrial workers	Workers with acute or chronic exposure to industrial chemicals
Other workers	Farmers
	Hairdressers
	Healthcare workers with specific activities (e.g., radiographers, anesthetists)
	Urban policemen
	Flight crew
	Cabin crew
	Swimming pool workers

People who live or work indoors	Teachers
	Students
	Office employees
	Housewives
	Construction workers
	House painters

People who might be exposed to toxic chemicals only once	Workers with exposure to pesticides
	Workers with exposure to drugs
	Victims of industrial accidents
	Victims of chemical accidents

Office workers	Office workers in tightly closed buildings

**Table 3:** Agents related to MCS.

(i) Organic solvents, paints, and lacquers for finishes (xylene, methylene chloride, petroleum distillates, glycol ethers, and trichloroethane)	
(ii) Pesticides (diazinon, azinphos-methyl [Guthion], and other organophosphates)	
(iii) Smoke and fumes from welding	
(iv) Metals (nickel, lead)	
(v) Various chemicals (formaldehyde, freon, ethanol, nitric acid, hydrochloric acid, and toluene)	
(vi) Powder and dust (wood, beet sugar)	
(vii) Food	
(viii) Certain diseases (scabies, herpes zoster)	
(ix) Perfume and air fresheners (shampoo, nail varnish and nail varnish remover, colognes, shaving lotions, various cosmetics, deodorants, etc.)	
(x) Furniture	
(xi) Paper	
(xii) New buildings	
